# IsoPepTracker: An interactive web application for peptide-driven isoform analysis

**DOI:** 10.1371/journal.pcbi.1014324

**Published:** 2026-06-03

**Authors:** Araf Mahmud, Chen Huang

**Affiliations:** 1 Department of Genetics, University of Alabama at Birmingham, Birmingham, Alabama, United States of America; 2 O’Neal Comprehensive Cancer Center, University of Alabama at Birmingham, Birmingham, Alabama, United States of America; The Pennsylvania State University, UNITED STATES OF AMERICA

## Abstract

Alternative splicing affects 95% of multi-exon genes, generating protein isoforms with distinct functions. While current alternative splicing analyses effectively identify splice events at the RNA level, they provide limited protein-level insight. To address this gap, we developed **IsoPepTracker** (https://www.isopeptracker.org), a user-friendly web application for analyzing and visualizing differential peptides across canonical and novel isoforms that are theoretically detectable by shotgun mass spectrometry-based proteomics. IsoPepTracker features four modules: Canonical Isoform Analysis, Novel Isoform Discovery, Peptide Sequence Search, and Alternative Splicing Analysis. Each module is tailored for distinct and complementary proteogenomics analyses. Users can input genes, novel cDNA sequences, peptides, or alternative splicing results to pinpoint peptides of interest and identify their associations with target genes or isoforms. We demonstrate the straightforward application of IsoPepTracker in proteogenomics through case studies. IsoPepTracker not only provides informative peptide signatures to understand the protein-level consequences of alternative splicing but also supplies peptide candidates for validation in shotgun proteomics.

## Author summary

Alternative splicing greatly expands the variety of proteins a single gene can produce. Yet while proteins drive nearly all biological functions, most splicing studies stop at the RNA level—making it hard to predict the actual changes in proteins. Here, we introduce **IsoPepTracker**, a user-friendly web tool that reveals the protein-level consequences of alternative splicing. Researchers can explore a gene list or upload their own data to identify unique peptides and determine which protein isoforms they belong to. By bridging RNA differences to their protein outcomes, IsoPepTracker makes it easier to understand the functional impact of splicing.

## Introduction

Alternative splicing (AS) affects ~95% of multi-exon human genes [1,2], enabling single genes to produce multiple protein isoforms through differential exon inclusion, intron retention, and alternative splice site usage [3]. The resulting isoforms can differ in enzymatic activity, subcellular localization, protein interactions, and stability [4,5]. Dysregulated splicing contributes to various diseases [6,7], making the identification of functionally relevant splice variants a critical research priority.

RNA sequencing (RNA-seq) provides a powerful platform for splice variant discovery through sophisticated computational approaches. Event-level AS tools like rMATS [8], MAJIQ [9], SplAdder [10], and SUPPA2 [11] identify and quantify AS events between experimental conditions. In comparison, transcript-level AS tools like StringTie [12], Trinity [13], and Cufflinks [14] reconstruct and quantify full-length mRNA isoforms across samples. These approaches routinely identify thousands of splice variants per experiment. However, transcript catalogs cannot reveal which splice variants are translated into proteins due to diverse post-transcriptional regulations [15,16]. For instance, nonsense-mediated decay might eliminate alternatively spliced RNAs [17,18], and translational efficiency and protein stability might vary dramatically between mRNA isoforms [19,20].

Mass spectrometry (MS) proteomics enables genome-wide profiling of protein identity and abundance. In bottom-up MS proteomics, which offers the highest throughput, proteins are digested with proteases into peptides and then fragmented to generate MS/MS spectra [21]. These spectra are matched to theoretical spectra from database-derived peptide candidates to identify peptide sequences. Proteins are subsequently inferred from these peptide sequences [22].

Compared to RNA-seq, the sensitivity of MS proteomics is much lower in covering whole protein sequences. Isoform-specific peptides spanning exon-exon junctions or originating from alternatively included exons, which are informative for identifying AS events, are usually lacking [23]. Therefore, it is important to prioritize these peptides to study AS events at the protein level. While traditional MS data analyses collapse protein isoforms into genes or protein groups for harmonized data quantification, novel tools like SEPepQuant [24] and IsoBayes [25] have recently been proposed to improve isoform-level inference. These methods use graph theory and Bayesian inference to assign ambiguous peptides to specific isoforms. However, these tools operate retrospectively from acquired proteomics data. There is an unmet need for identifying and prioritizing key peptides associated with AS events, especially when the knowledge of these events is expanded by more advanced long-read sequencing. Theoretical characterization of these peptides will not only help systematically evaluate the power of current MS data for AS studies, but also provide candidates for peptide-targeted MS experiments, such as selective or Parallel Reaction Monitoring (SRM or PRM) MS.

Moreover, protease cleavage specificity is an important factor impacting junction peptide detection. Trypsin, the predominant protease used in MS proteomics, cleaves specifically after lysine (K) and arginine (R). However, approximately 25% of human exon boundaries naturally terminate with K or R codons, thus reducing the generation of junction-spanning peptides [26]. Recent deep proteomics studies using multiple proteases with complementary cleavage specificities have demonstrated substantial improvements in splice variant detection [27,28]. However, despite the critical importance of enzyme selection, a computational framework to predict which proteases will generate detectable junction peptides for specific splice variants is currently lacking. Although tools like PeptideCutter [29] and Rapid Peptides Generator [29] can predict cleavage sites for protein sequences, they do not provide integrated analysis to annotate enzyme specificity in the context of AS.

In this study, we developed IsoPepTracker, a multifaceted and interactive web tool to bridge the gap between splicing variants and their peptide signatures. The central function of IsoPepTracker is to identify and visualize key peptides that can distinguish isoforms and inform AS events. The isoforms of interest can be queried by gene, derived from long-read sequencing, or resulting from differential AS events. Moreover, IsoPepTracker’s analysis and visualization can be customized for protease enzyme selections, enabling users to inspect optimal enzyme choices for specific AS event detection.

## Results

### Overview of IsoPepTracker

The primary goal of IsoPepTracker is to enable researchers to predict, visualize, and characterize the protein-level isoform diversity that can be detected by shotgun MS, which remains the only high-throughput technique for profiling global proteomics. To highlight the importance of peptide-level annotation over traditional exon level annotation, we performed a genome-wide simulation of protein translation and MS protease digestion. Based on fully digested proteolytic peptides without missed cleavage sites, which are the dominant species in proteomic data (S1 Fig), we demonstrated that alternative splicing (AS) events at the RNA level are not always translated into differential peptides across protein isoforms. This discrepancy arises not only because some AS events occur in non-coding regions but also due to the inherent peptide size requirement of MS-based proteomics (S2 Fig). Notably, the protein sequence coverage provided by these peptides varies significantly across different proteases, highlighting the importance of enzyme selection in detecting protein isoforms (S3 Fig). Consequently, the core principle of IsoPepTracker is to facilitate the theoretical translation of genome-wide AS events into isoform-specific peptide signatures and improve the design of MS proteome strategies for specific isoforms and AS events of interest.

**Fig 1:**
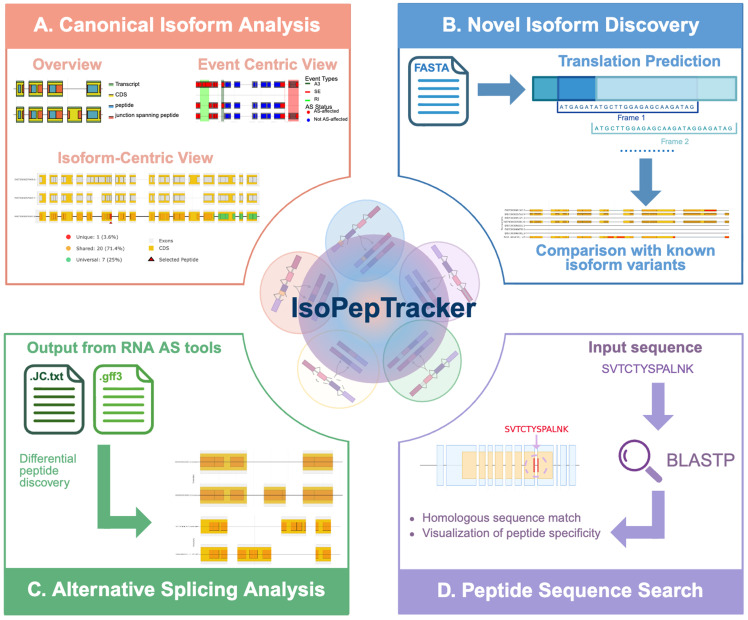
IsoPepTracker workflow. (A) *Canonical Isoform Analysis* module analyzes and visualizes shared or isoform-specific peptides for canonical genes and transcripts. (B) *Novel Isoform Discovery* module predicts translations and compares them with known isoform variants. (C) *Alternative Splicing Analysis* module identifies and annotates differential peptides generated from splicing events. (D) *Peptide Sequence Search* module identifies the isoform-specificity of queried peptides. Created in BioRender. Mahmud, A. (2026) https://biorender.com/f73lc72.

IsoPepTracker is organized into four main functional modules, each addressing specific analytical needs in the proteogenomics workflow (Fig 1). The *Canonical Isoform Analysis* module provides comprehensive analysis of annotated transcript isoforms from the reference database and can be queried by gene IDs or symbols. The *Novel Isoform Analysis* module enables the characterization of novel transcript sequences discovered through long-read sequencing, de novo assembly, or results from isoform-level AS tools. The *Alternative Splicing Analysis* module focuses on integration with external AS detection tools and comparative evaluation of specific splicing events. Finally, the *Peptide Sequence Search* module provides integrated sequence similarity searching of peptides and maps them to their transcript origins.

IsoPepTracker’s visualization engine generates comprehensive multi-layered genomic views that integrate transcript structures with peptide-level information. The platform displays exon-intron structure alongside mapped peptides, enabling researchers to visualize the relationship between genomic structure and proteomic detectability. Each peptide is positioned according to its genomic coordinates, creating an intuitive representation of proteolytic coverage across splicing variants. The interactive interface allows users to explore individual peptides through hover functionality, revealing amino acid sequences and corresponding genomic positions. All visualizations and peptide sequences can be downloaded as vectorized figures (e.g.,.pdf or.svg) and text files (.txt), respectively.

### Canonical isoform analysis

**Fig 2:**
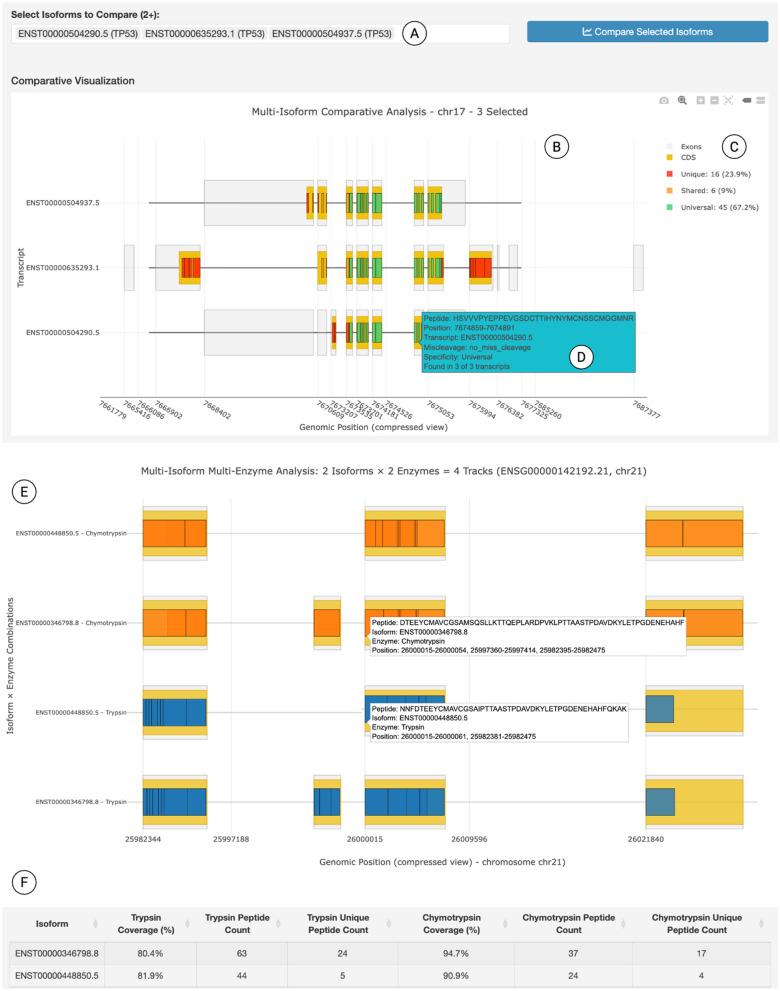
Isoform-centric visualization for canonical isoforms. (A-D) The upper panel shows a track plot displaying the transcript structure and differential peptides. For a given gene (e.g., TP53), users can select any combination of its isoforms (A) for visualizing the transcript and mapped peptides (B). These are annotated with a legend indicating peptide specificity (C) and an annotation box with detailed peptide information upon hovering (D). (E) The middle panel compares trypsin and chymotrypsin digestion for two transcript variants. Orange and blue tracks represent chymotrypsin- and trypsin-digested peptides, respectively. (F) The lower panel shows a table from the portal quantifying peptide coverage and unique peptide counts for each enzyme. All interface elements shown are original components of the IsoPepTracker portal; no third-party browser graphics or copyrighted icons are included.

**Fig 3:**
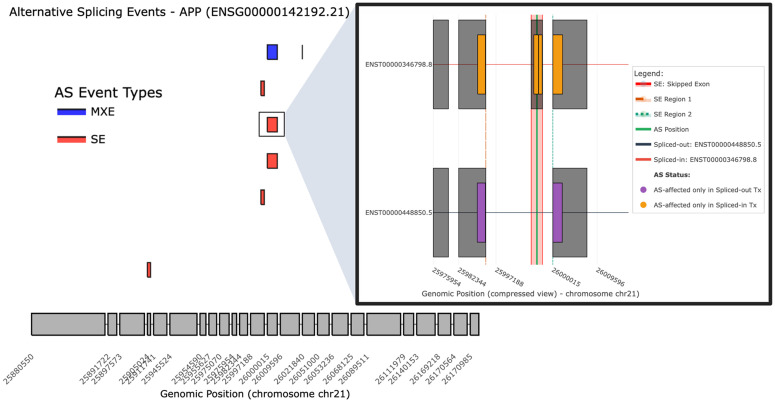
AS event-centric visualization for canonical isoforms. For a given gene (e.g., *APP*), the portal identifies and visualizes all the AS events related to its isoforms. The inset highlights differential peptides for the user-selected events.

One question frequently pursued in isoform-level proteogenomics studies is how to identify key peptides that can distinguish specific isoforms or characterize AS events. The *Canonical Isoform Analysis* module provides two complementary analysis modes to achieve this goal. First, the isoform-centric tab allows users to select an isoform combination for a gene of interest and obtain the distinguishing peptides specific to that selection (Fig 2A). This analysis can be customized by specifying proteomic parameters, including the proteolytic enzyme, the size range of peptide, and the number of allowed missed cleavages. The platform generates an informative track plot displaying the transcript structure with mapped peptide locations (Fig 2B), color-coded by their specificity levels—CDS regions, unique peptides, shared peptides, and universal peptides—with a legend indicating peptide statistics (Fig 2C) and a tooltip function revealing detailed peptide information upon hovering (Fig 2D). Moreover, users can compare different enzymatic digestions in identifying isoform-specific peptides for a given isoform, enabling a direct comparison between proteases such as chymotrypsin and trypsin with their respective peptides (Fig 2E and 2F). Second, the event-centric tab allows users to analyze and visualize peptides resulting from AS events that account for the isoform diversity (Fig 3). For each gene-AS event combination, the portal identifies and annotates the isoforms and differential peptides that are generated by the AS. Together, the *Canonical Isoform Analysis* module enables users to identify informative peptides and optimal enzymes for their genes and isoforms of interest. Such analysis is useful for designing targeted MS assays (e.g., SRM and PRM) or for selecting the most appropriate enzymes to distinguish target isoforms during experimental design or dataset selection during secondary proteomics data analysis.

### Novel isoform analysis

**Fig 4:**
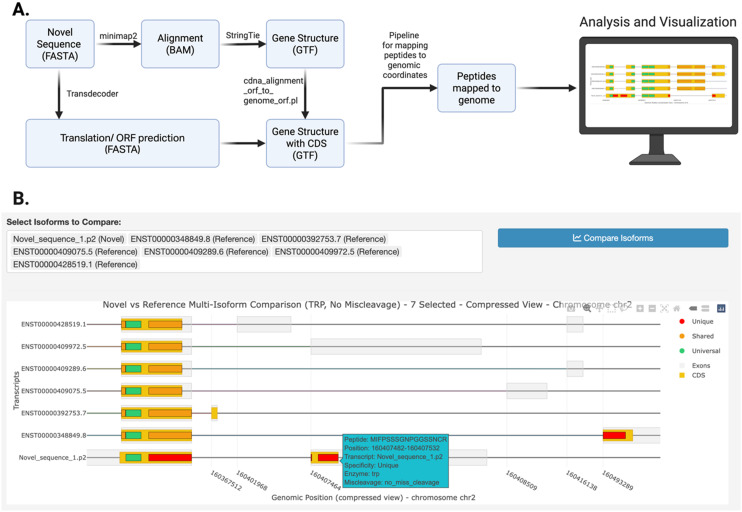
Identification and annotation of unique peptides for novel isoforms. (A) Workflow for novel isoform alignment and differential peptide discovery. Created in BioRender. Mahmud, A. (2026) https://biorender.com/r5eyj2x. (B) Multi-isoform comparative analysis featuring the LRS-identified novel isoform of *RBMS1*. The visualization highlights the isoform-specific candidate peptide “MIFPSSSGNPGGSSNCR”, which can be used to verify its expression at the protein level. All interface elements shown are original components of the IsoPepTracker portal; no third-party browser graphics or copyrighted icons are included.

Besides canonical isoforms annotated in the reference transcriptome, novel isoforms can be generated from RNA-seq data through *de novo* assembly (e.g., Trinity [30]) or isoform-level quantification tools (e.g., StringTie [12], Cufflinks [14]). Moreover, the recently developed long-read sequencing (LRS) has demonstrated its power in identifying transcripts undetectable by conventional RNA-seq. The *Novel Isoform Analysis* module is designed for users who have a transcript sequence from any of the abovementioned sources and aim to identify its unique peptides compared to protein sequences from canonical reference databases. The input is a transcript sequence in FASTA format, and the platform performs sequence alignment, open reading frame (ORF) prediction, and peptide mapping and annotation (Fig 4A and S1 Note).

Fig 4B demonstrates a case study using a novel isoform sequence of *RBMS1* from a recent LRS study [31]. The module first mapped the sequence to the reference *RBMS1* isoform set and then performed a multi-isoform comparative analysis, where the novel isoform was aligned with five reference transcripts that share similar transcript structure. Subsequently, through ORF prediction, *in silico* peptide digestion, and multi-isoform comparison, an isoform-specific peptide (“MIFPSSSGNPGGSSNCR”) can be identified for this novel isoform.

Therefore, the integration of novel and reference data within a single analytical framework in this module will facilitate the translation of novel transcripts into proteoform variants, expanding our understanding of proteome diversity beyond traditional annotation databases.

### Alternative splicing analysis

**Fig 5:**
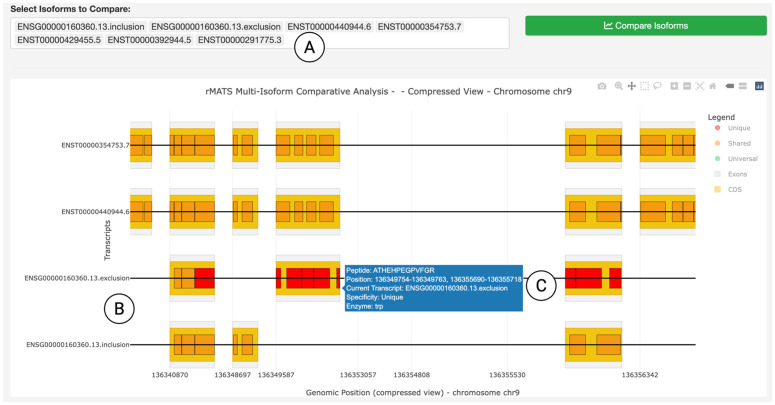
Peptide-level AS outcome from external AS tools. (A) An input box for specifying inclusion, exclusion, and canonical isoforms for comparison. (B) Visualization of inclusion and exclusion isoforms along with canonical isoforms. (C) A tooltip feature that reveals detailed peptide information upon hovering. All interface elements shown are original components of the IsoPepTracker portal; no third-party browser graphics or copyrighted icons are included.

Besides isoform-level AS identification, another class of external AS tools focuses on characterizing AS events, such as exon skipping, intron retention, alternative 5’ or 3’ splice sites [8,10]. Although these events delineate dynamic RNA splicing changes at exon-exon junctions across biological conditions, inferring their protein-level consequences is not straightforward. To address this challenge, IsoPepTracker provides an *Alternative Splicing Analysis* module that translates AS events into isoform-level comparisons, identifies differential peptides, and provides relevant peptide annotation and visualization. Fig 5 shows a case study demonstrating the platform’s ability to process splicing events from external sources and visualize their peptide-level consequences (S1 Note). The interface allows users to specify AS events identified by rMATS [8] to generate inclusion and exclusion isoforms, as well as other structurally similar isoforms for comparison through an input box (Fig 5A). The portal then generates a visualization that displays differential peptides caused by the AS event and annotates their isoform-specificity (Fig 5B). A tooltip function that reveals detailed peptide information upon hovering (Fig 5C), showing peptide sequences, positions, and associated transcript identifiers. This visualization demonstrates how the rMATS-derived splicing events are translated into isoform-level comparisons, with the highlighted peptide regions revealing molecular signatures, thereby providing actionable targets for experimental validation, such as an epitope for antibody design or a peptide target for SRM MS.

### Integrated peptide search engine

**Fig 6:**
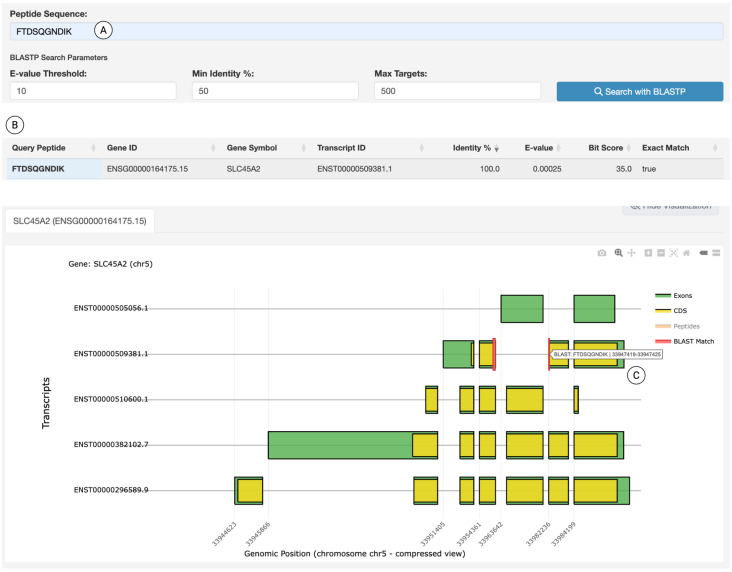
Peptide search and isoform-specificity analysis. (A) An input box for specifying a query peptide (e.g., FTDSQGNDIK). (B) BLAST results displaying information on matched transcripts. (C) Track visualization mapping the query peptide to the transcript, alongside comparisons with other structurally related transcripts. All interface elements shown are original components of the IsoPepTracker portal; no third-party browser graphics or copyrighted icons are included.

Finally, the *Peptide Sequence Search* module provides an integrated peptide-centric search and annotation pipeline. In proteogenomics, a peptide-centric analysis often starts with a peptide of interest—such as an immunogenic peptide that could serve as an antigen for cancer immunotherapy—and aims to determine its specificity across different protein isoforms. In this module, the user inputs a peptide sequence, and the platform retrieves all isoforms that either encode this peptide or lack it but share a similar transcript structure. Fig 6 demonstrates a case study using a recently identified immunogenic peptide FTDSQGNDIK [32] as input and setting up customizable BLASTP parameters (Fig 6A and S1 Note). Users can customize the search by specifying digestion parameters, such as the non-enzymatic cleavage pattern, which is characteristic of the immunopeptidome. The search returns matched transcripts with gene IDs, identity scores, and E-values (Fig 6B). The portal maps the peptide to the *SLC45A2* gene’s transcript ENST00000509381.1. A track plot is generated to visualize the peptide localization and isoform specificity (Fig 6C). When combined with gene expression profiles, the antigen’s transcript specificity revealed by the portal is valuable for determining which patients or tissue types are responsive to corresponding vaccines or T cells.

## Discussion

The rapid identification of transcriptomic diversity, driven by advances in RNA-seq and long-read sequencing, has created a significant gap between the cataloging of splice variants and the validation of their protein-level expression. Identifying which novel isoforms are translated and how AS events impact the proteome remains a major challenge. To address this, we have developed IsoPepTracker, an interactive, multifaceted web tool designed to facilitate the translation of RNA-level AS identification to their corresponding peptide signatures.

We have demonstrated that IsoPepTracker successfully integrates and analyzes data from various sources. The platform performs *in silico* proteolytic digestions and maps the resulting peptides to their genomic origins. This provides clear, interactive visualizations of isoform-specific, shared, and universal peptides, moving beyond simple gene-level comparisons. In addition, the comparison of peptide-level consequences across different proteases empowers researchers to strategically select an optimal enzyme for detecting a specific splice variant of interest.

By translating complex AS events into concrete, prioritized peptide candidates, IsoPepTracker serves as a powerful hypothesis-generation and experimental design tool. It provides researchers with actionable, sequence-level targets for validating protein expression, whether through targeted mass spectrometry (SRM/PRM) or for the development of isoform-specific antibodies. This function is critical for the field of proteogenomics, offering a robust method to confirm that transcriptomic variants are not just artifacts but are translated into functionally relevant proteins.

On the other hand, IsoPepTracker’s predictive power is currently limited to theoretical peptide sequences. Real-world detection depends on factors the tool does not yet model, such as isoform-specific expression levels, protein stability, post-translational modifications (PTMs), and the ionization efficiency of a given peptide. Furthermore, the accuracy of the novel isoform and AS event analyses is contingent upon the quality of the input data, such as the completeness of long-read assemblies or the precision of AS event coordinates. Future development will integrate IsoPepTracker with public proteomics repositories and spectral libraries, allowing users to cross-reference predicted peptides with existing experimental evidence. For instance, incorporating proteomics resources from large-scale consortia, such as CPTAC [33] or CCLE [34], will provide experimentally derived peptide data across diverse tissue types. Additionally, incorporating PTM predictions and more sophisticated models of peptide “detectability” will further refine the prioritization of candidates.

In summary, IsoPepTracker provides an accessible and essential framework for exploring the functional proteome, facilitating a deeper understanding of how alternative splicing shapes protein diversity in health and disease.

## Materials and methods

**Generation of Splice-Aware Peptide Databases** Comprehensive peptide databases were generated by *in silico* proteolytic digestion of all 106,143 human proteins annotated from GENCODE v38 [35], using the cleaver R package [36] with six proteases (trypsin, LysC, LysN, AspN, chymotrypsin, and GluC). Two databases were created to reflect experimental conditions: one with full digestion and the other allowing up to two missed cleavages. By default, peptides were filtered to retain sequences between 6 and 60 amino acids (AA), representing the optimal detection range for mass spectrometry (MS)-based proteomics; however, these parameters are fully customizable by the user. For each peptide, AA positions were converted to CDS nucleotide coordinates and then mapped to genomic reference coordinates using customized R script.

The following Bioconductor packages were used to build genomic/transcriptomic data infrastructure: rtracklayer [37] for GTF/GFF file processing, GenomicRanges [38] and IRanges [39] for interval operations and coordinate transformations, BSgenome.Hsapiens.UCSC.hg38 [40] for reference genome access, and Biostrings [40] for sequence retrieval and translation.

**AS event-centric View for Canonical Isoforms** AS events in protein-coding genes were identified from the GENCODE human v38 gene annotation using SUPPA2 [11]. For each identified splicing event (i.e., skipped exons, retained introns, mutually exclusive exons, alternative 3’ splice sites, and alternative 5’ splice sites), combined genomic ranges were created encompassing all alternatively spliced regions. The GenomicRanges::findOverlaps function was employed to identify peptides whose genomic coordinates intersected with alternative splicing regions by at least one nucleotide. Peptide classification was performed by comparing peptide presence between inclusion and exclusion transcript variants using set operations.

**Isoform Specificity Analysis** Peptide categorization was performed by calculating the number of transcripts containing each peptide sequence and assigning categories based on presence patterns: peptides found in only one transcript were labeled as *unique*, those in a subset of transcripts as *shared*, and those in all transcripts as *universal*. Multi-isoform comparative analysis was implemented using matrix operations to calculate Jaccard similarity coefficients between peptide sets. Coverage metrics were computed using the IRanges::reduce function to merge overlapping peptide positions and calculate the proportion of protein sequence covered.

**Novel Isoform Discovery Pipeline** Novel isoform analysis is implemented as an automated pipeline integrating multiple bioinformatics tools. The user-submitted FASTA sequences are processed through TransDecoder (https://github.com/TransDecoder/TransDecoder) to predict open reading frames (ORFs) based on sequence composition and codon usage bias. In parallel, the sequence input is mapped to the GRCh38 reference genome using minimap2 [41] with splice-aware parameters (-ax splice mode). StringTie2 [12] is applied to the minimap2 output to reconstruct transcript structure and generate GTF annotations. Transcript structure resulting from the mapping is integrated with the ORF prediction using cdna_alignment_orf_to_genome_orf.pl from TransDecoder. Finally, the six-enzyme proteolytic digestion protocol is applied using the same cleaver package functions as for annotated transcripts, and peptides are mapped to genomic coordinates as described above.

**Peptide-centric View** Peptide sequence search was implemented using BLASTP from the NCBI BLAST+ suite. A BLAST database was constructed from GENCODE v38 protein sequences using makeblastdb. User-submitted peptide sequences are searched against the database with configurable parameters including an E-value threshold, identity cutoff, and maximum target sequence count. Peptides are mapped to genomic coordinates for the isoforms that have matched sequence. The matched regions are displayed as red highlights on the isoforms. When a peptide matches multiple genes, each gene is displayed in a separate tab showing its isoforms with the highlighted peptide regions.

**Alternative Splicing Analysis Module** The Alternative Splicing Analysis Module currently supports the results from two popular event-level AS tools: rMATS [8] and SplAdder [10]. The tabular output file containing genomic coordinates for five splicing event types (SE, MXE, A3SS, A5SS, RI) is parsed to extract alternative exon coordinates and translational phase information from GENCODE annotations. Strand-specific sequences are extracted from the hg38 genome sequence using BSgenome.Hsapiens.UCSC.hg38::getSeq and translated into AA sequences using Biostrings::translate. The six-enzyme proteolytic digestion protocol is applied, and peptides are mapped to genomic coordinates similarly as described above.

**Visualization Implementation** The visualization of transcript structure and peptide alignment is implemented using plotly [42] and ggplot2 [43]. Exons are rendered as rectangular elements positioned according to genomic coordinates extracted from GTF data, with CDS regions overlaid as narrower rectangles to distinguish coding from non-coding sequences. Peptides are mapped on top of the transcript dynamically based on peptide overlap to prevent visual occlusion. Each peptide rectangle spans its genomic coordinates with height adjusted to create a stacked layout. Custom hover text for each peptide displays AA sequence, genomic position, enzyme source, and isoform specificity.

**Tool Accessibility** IsoPepTracker is implemented as an open-source R Shiny application (with JavaScript-powered user interface) at http://www.isopeptracker.org. The source code is available at https://github.com/HuangLabAtUAB/IsoPepTracker at under an MIT license. For all the modules and submodules, we have provided detailed descriptions of their functionalities and usage via an online document: https://isopeptracker-docs.readthedocs.io/en/latest

## Supporting information

S1 Fig Barplots illustrating the distribution of peptides with and without missed trypsin cleavage sites. (TIFF)

S2 Fig Protein-level detectability by MS proteomics for each type of alternative splicing (AS) event. Undetectability can be attributed to either a lack of peptides resulting from the AS event (red) or peptide lengths that fall outside the MS-detectable range (yellow).
(TIFF)

S3 Fig Box plots showing the percentage of sequence coverage by MS-detectable peptides. Each box plot represents peptides generated by a specific protease; the mean and median coverage values across all proteins are indicated above each plot.
(TIFF)

S1 Note Details for reproducing the figures in the manuscript. (DOCX)
