# Supplementary figures and images for: IsoPepTracker: An interactive web application for peptide-driven isoform analysis
Source: PLoS Comput Biol. 2026 Jun 3;22(6):e1014324. doi: 10.1371/journal.pcbi.1014324 (PMC13232816; doi:10.1371/journal.pcbi.1014324)

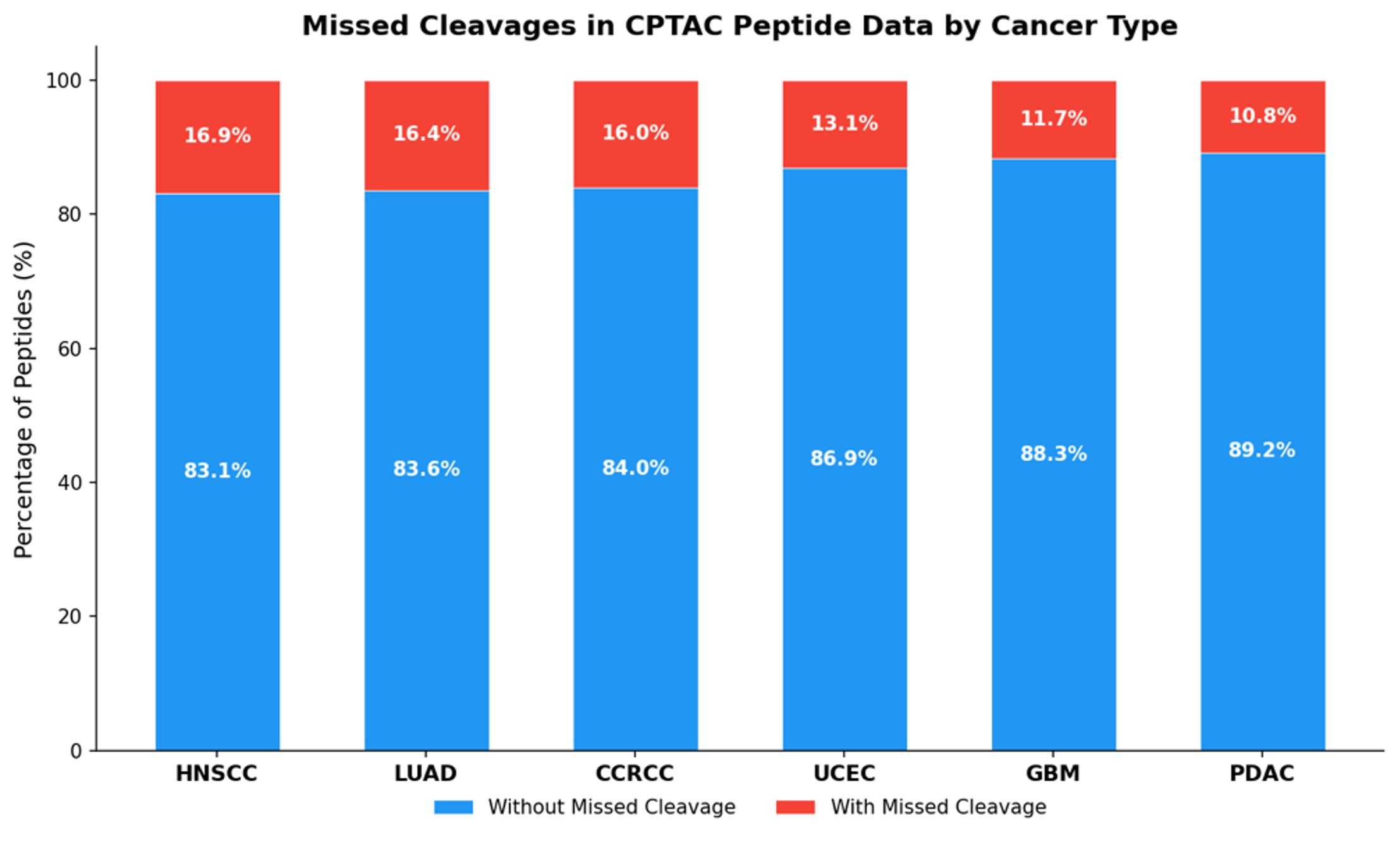

Supplement: S1 Fig — (TIFF) [file pcbi.1014324.s001.tiff]

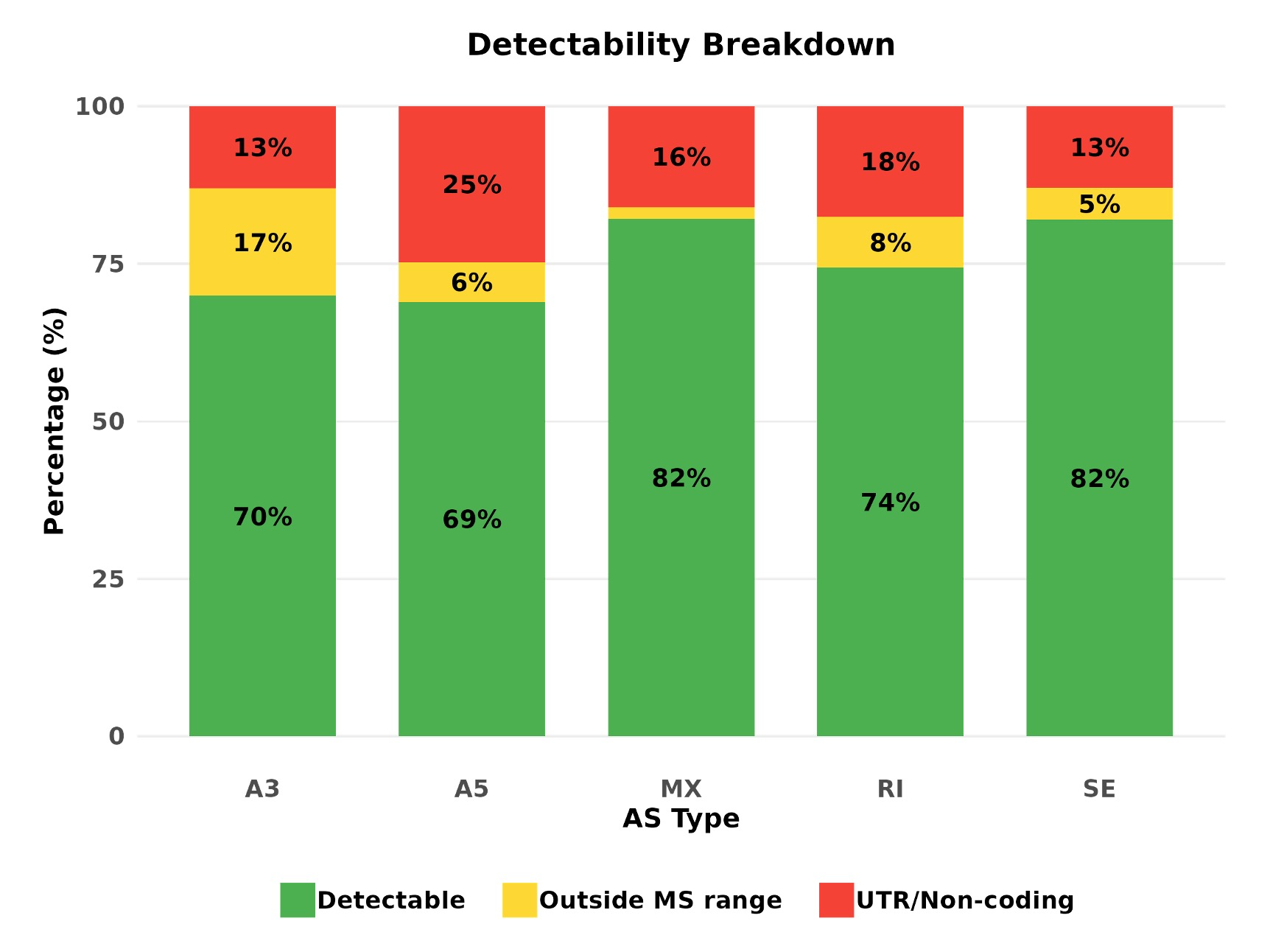

Supplement: S2 Fig — Undetectability can be attributed to either a lack of peptides resulting from the AS event (red) or peptide lengths that fall outside the MS-detectable range (yellow). (TIFF) [file pcbi.1014324.s002.tiff]

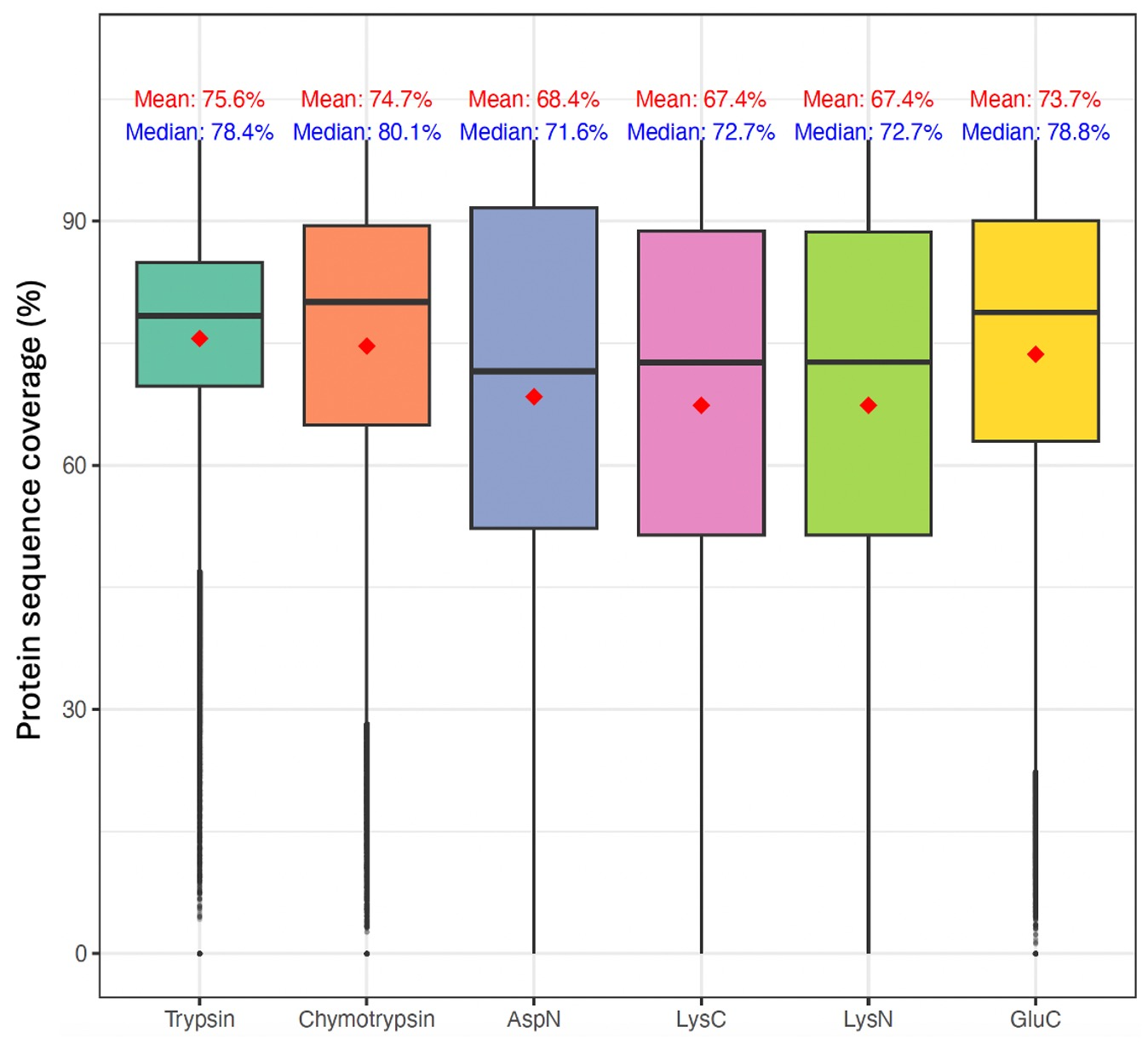

Supplement: S3 Fig — Each box plot represents peptides generated by a specific protease; the mean and median coverage values across all proteins are indicated above each plot. (TIFF) [file pcbi.1014324.s003.tiff]
