# Supplementary material for: IsoPepTracker: An interactive web application for peptide-driven isoform analysis
Source: PLoS Comput Biol. 2026 Jun 3;22(6):e1014324. doi: 10.1371/journal.pcbi.1014324 (PMC13232816; doi:10.1371/journal.pcbi.1014324)
Supplement: S1 Note — (DOCX) [file pcbi.1014324.s004.docx]

**S1 Note**

All figures in this manuscript were generated using IsoPepTracker (https://isopeptracker.org). **The following describes the procedures used to generate each figure.**

**Figure 2** was generated using the Canonical Isoform Analysis module. The *TP53* gene was selected in the analysis parameters, with trypsin specified as the protease (no missed cleavages allowed). Within the isoform-centric tab, the compare multiple isoforms functionality was utilized. Three TP53 transcript variants (ENST00000504290.5, ENST00000635293.1, and ENST00000504937.5) were selected from the dropdown menu. For the second part of Figure 2, the APP gene was selected in the analysis parameters. Through the multi-isoform multi-protease comparison feature in the isoform-centric tab, two APP isoforms (ENST00000348850.3 and ENST00000348798.8) were selected from the dropdown menu and chymotrypsin and trypsin enzymes were selected as proteases, with parameters allowing up to 2 missed cleavages.

**Figure 3** was created using the *event-centric tab* with the *APP* gene selected. The specific splicing event (ENSG00000142192.21;SE:chr21:25982477-25997360:25997416-26000015:-) was selected from the table by clicking on the event. After the event was selected, "Spliced-out: ENST00000448850.5" versus "Spliced-in: ENST00000346798.8" was selected from the dropdown menu.

**Figure 4** was generated using the *Novel Isoform Discovery* module. A FASTA sequence file containing the novel isoform sequence was uploaded (<https://github.com/HuangLabAtUAB/IsoPepTracker/blob/main/test/novel_isoform_analysis/novel_RBMS1.fa>) and translated. The “Novel_sequence_1.p2” translation was selected for analysis. The *RBMS1* gene was selected to load the reference isoforms for comparison.

**Figure 5** was generated using the *Alternative Splicing Analysis* module's rMATS analysis functionality. The mutually exclusive exon (MXE) event type was selected, and then an rMATS output file containing events was uploaded (<https://github.com/HuangLabAtUAB/IsoPepTracker/blob/main/test/rmats_test/MXE_test.txt>), and the target event row was selected from the table. The analysis generated corresponding inclusion (ENSG00000160360.1.inclusion) and exclusion (ENSG00000160360.13.exclusion) isoforms, which were displayed alongside canonical reference isoforms (ENST00000440944.6, ENST00000354753.7, ENST00000429455.5, ENST00000392944.5, ENST00000291775.3).

**Figure 6** was generated using the *Peptide Sequence Search* module. A query peptide "FTDSQGNDIK" was submitted using default BLAST parameters in the peptide sequence search module. The generate visualization button was clicked to show the visualization.
